# Bone marrow aspirate concentrate versus platelet-rich plasma for treating knee osteoarthritis: a one-year non-randomized retrospective comparative study

**DOI:** 10.1186/s12891-021-04910-5

**Published:** 2022-01-03

**Authors:** Abed El-Hakim El-Kadiry, Carlos Lumbao, Natasha Salame, Moutih Rafei, Riam Shammaa

**Affiliations:** 1 Laboratory of Thrombosis and Hemostasis, Montreal Heart Institute, Research Center, Montreal, QC Canada; 2 Department of Biomedical Sciences, Université de Montréal, Montreal, QC Canada; 3 Canadian Centres for Regenerative Therapy, Toronto, ON Canada; 4 Department of Pharmacology and Physiology, Université de Montréal, Montreal, QC Canada; 5 Department of Microbiology, Infectious Diseases and Immunology, Université de Montréal, Montreal, QC Canada; 6 Molecular Biology Program, Université de Montréal, Montreal, QC Canada; 7 Department of Microbiology and Immunology, McGill University, Montreal, QC Canada; 8 Department of Family and Community Medicine, University of Toronto, Toronto, ON Canada

**Keywords:** Knee, Osteoarthritis, Bone marrow aspirate concentrate, Platelet-rich plasma, Mesenchymal stromal cells, Visual analogue scale, Knee injury and osteoarthritis outcome score, Western Ontario and McMaster universities arthritis index

## Abstract

**Background:**

Knee osteoarthritis (OA) is a debilitating condition affecting human body biomechanics and quality of life. Current standard care for knee OA leads to trivial improvement and entails multiple adverse effects or complications. Recently, investigational cell therapies injected intra-articularly, such as bone marrow aspirate concentrate (BMAC) and platelet-rich plasma (PRP), have shown safety and therapeutic potency providing patients with pain relief. In the current retrospective comparative study, we investigated the differences in pain and functional improvements in patients with symptomatic knee OA receiving intra-articular injections of BMAC vs PRP.

**Methods:**

Pain and functionality scores were measured at baseline and at different time points post-injection over 12 months, using 3 self-administered, clinically validated questionnaires: the visual analogue scale (VAS) for assessing pain intensity, the knee injury and osteoarthritis outcome score (KOOS) for evaluating functionality and knee-related quality of life, and the Western Ontario and McMaster Universities Arthritis Index (WOMAC) for evaluating physical function. The repeated-measures general linear model with Sidak test for pairwise comparisons was used to investigate the influence of the treatment on the score evolution within groups (between baseline and each time point) and between groups (overall).

**Results:**

The BMAC group (*n* = 26 knees) significantly improved in VAS, KOOS, and WOMAC scores between baseline and 12 months (57.4, 75.88, and 73.95% mean score improvement, respectively). In contrast, the PRP group (*n* = 13 knees) witnessed nonsignificant improvement in all scores. BMAC, in comparison to PRP, induced significant improvement in outcomes by 29.38% on the VAS scale, 53.89% on the KOOS scale, and 51.71% on the WOMAC scale (*P* < .002, *P* < .01, *P* < .011, respectively).

**Conclusions:**

Intra-articular autologous BMAC injections are safe, effective in treating pain, and ameliorate functionality in patients with symptomatic knee OA to a greater extent than PRP injections.

**Graphical abstract:**

Intra-articular autologous BMAC therapy is safe and provides more relief to patients with symptomatic knee osteoarthritis compared to PRP therapy.
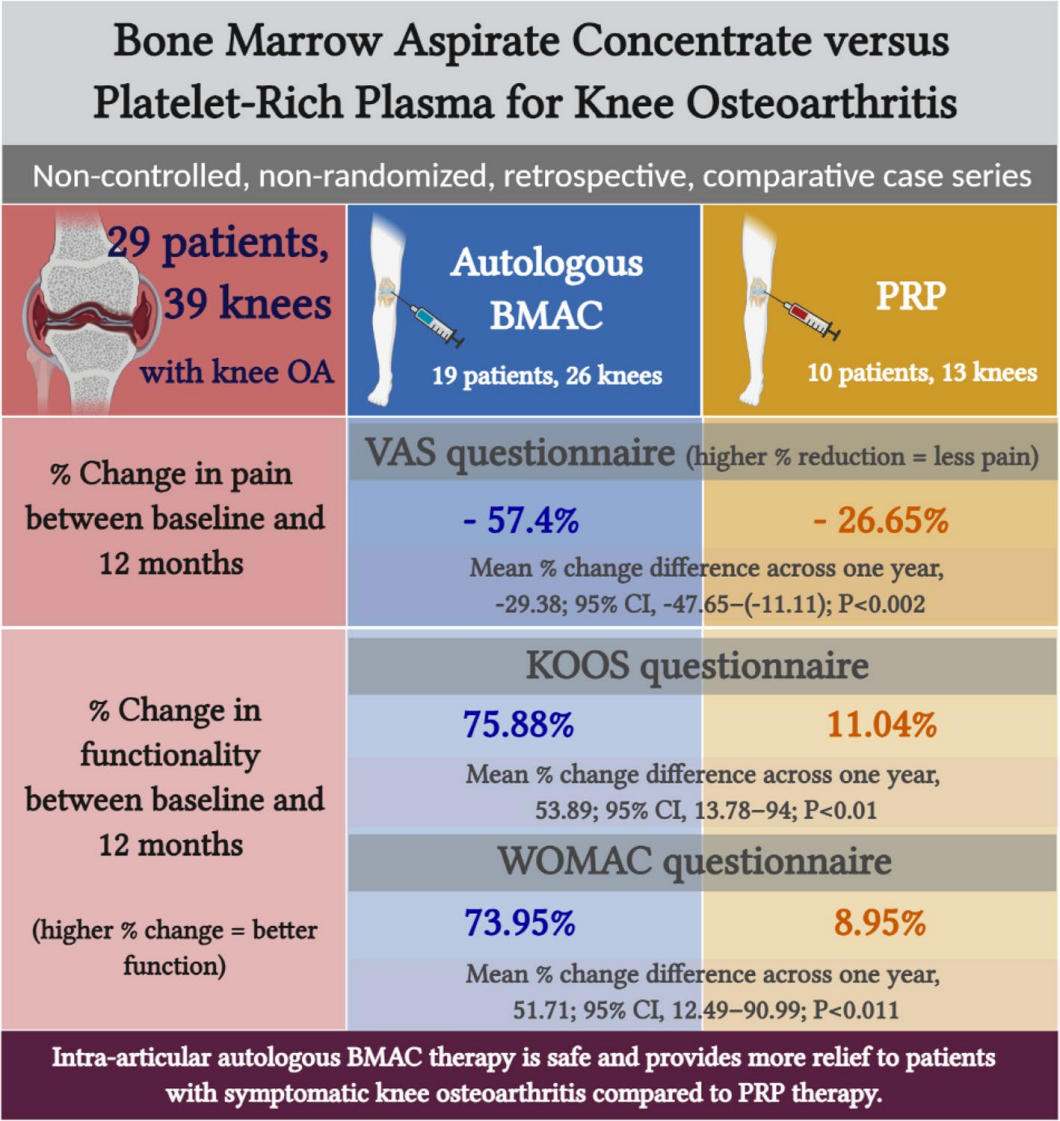

**Supplementary Information:**

The online version contains supplementary material available at 10.1186/s12891-021-04910-5.

## Introduction

Osteoarthritis (OA) is a degenerative joint disease impeding human body biomechanics and resulting in poor quality of life [1]. In Canada, 219,000 new cases were diagnosed with OA between 2016 and 2017 [2], with a sharp increase in OA patient numbers expected in the next 30 years [3]. OA of the knee is very frequent and results from the progressive degeneration of articular cartilage either idiopathically or following structural changes or trauma [4]. This causes joint pain and inflammation resulting in reduced joint range of motion (ROM) and mobility [5].

Standard care for knee OA includes conservative therapies or surgery [6]. Despite their affordability, management of pain, and/or delay of cartilage degeneration, conservative approaches like physiotherapy, analgesics, and intra-articular corticosteroid or hyaluronic acid (HA) injections have short-term efficacy and are still unproven to modify the disease [7]. Although surgery is recommended for end-stage knee OA, postoperative complications, cost, and lack of solid trial evidence remain problematic [8, 9]. In addition to relieving symptoms, newer investigational intra-articular therapies including cell therapies modalities are proposed to reverse the underlying pathological processes of knee OA [10, 11].

Platelet-rich plasma (PRP) is an autologous blood component with high concentrations of platelets– megakaryocyte-derived fragments, whose secretome promotes cartilage repair in vitro and in vivo [12]. In clinical studies, PRP injections in symptomatic patients with knee OA have demonstrated safety and better pain and mobility outcomes up to 12 months after treatment [13]. However, further clinical data are imperative to strengthen the evidence of their efficacy [14].

Another attention-drawing cell therapy is bone marrow aspirate concentrate (BMAC) [15]. The advantage of this therapy is its composition of multiple cell fractions including platelets, monocytes, and mesenchymal stem cells (MSCs)—the latter notorious for their multi-lineage differentiation potential and secretome-mediated regenerative effects [16, 17]. Clinically, BMAC and MSCs have demonstrated a promising therapeutic potential in multiple orthopedic conditions [18], including spinal OA [19] and knee OA [10, 17, 20]. However, the quality of clinical evidence corroborated by literature remains low [15, 21].

Backed by in vivo findings [22], few clinical studies have hitherto compared the functional and/or biological outcomes of PRP vs BMAC/MSC therapy in knee OA [23]. Therefrom, conflicting data have emerged mandating further investigations [24]. In this non-randomized comparative retrospective study, we sought to enhance available clinical knowledge by investigating potential differences in improvement on pain and functionality scales between PRP- and BMAC-treated patients. Outcomes were measured over 12 months at different time points using 3 self-administered, clinically validated questionnaires: i) the visual analogue scale (VAS) for assessing pain intensity on a scale of 0–10 cm [25–27], ii) the knee injury and osteoarthritis outcome score (KOOS) for evaluating symptoms, stiffness, pain, physical function (daily living and sports/recreational activities), and quality of life on a scale of 0–100 (100 = best function) [28, 29], and iii) the Western Ontario and McMaster Universities Arthritis Index (WOMAC) for evaluating symptoms, stiffness, pain, and daily function on a scale of 0–100 (100 = best function) [30–32].

## Materials and methods

### Study design and interventions

The herein research was conducted according to the World Medical Association Declaration of Helsinki. Conforming with the local legislative and procedural institutional obligations concerning the retrospective study nature, ethical review and approval was not mandated, similar to our recent spinal OA research [19]. Informed consent forms as a written expression of patient voluntary participation were obtained. Patients also consented to the anonymous publishing of collected data. From a single center, 30 patients diagnosed with symptomatic knee OA and meeting the selection criteria (Table 1) were recruited between September 2016 and July 2018 after being referred by their primary care physicians. Diagnosis was confirmed based on history, physical examination, and diagnostic imaging using X-rays with or without MRIs. All participants were well informed about the study objectives, associated risks and benefits, and treatment alternatives before and during the consent process. Patients received in their osteoarthritic knees either BMAC (*n* = 27 knees of 20 patients) or PRP (*n* = 13 knees of 10 patients) injections as delineated in the supplementary material (see Additional file 1: Tables 1–2).

**Table 1:** Selection criteria

Inclusion criteria	Exclusion criteria
18 years of age or older	Diagnosis of rheumatoid arthritis or other lower extremities-affecting musculoskeletal diseases
Clinically and/or radiologically confirmed diagnosis of knee OA (KL grade 1–4) in the past 6 months	Widespread pain
Knee pain (≥2 on VAS scale) lasting for 6 months or longer	Cancer
Willingness to discontinue analgesic medication for 48 h prior to each pain assessment	Knee surgery in the previous 6 months
	Arthrocentesis or intra-articular conservative therapy injections in the last 3 months
	History of stem cell knee injections
	Inability to provide informed consent

*KL* Kellgren Lawrence, *OA* Osteoarthritis, *VAS* Visual analogue scale

### BMAC preparation

As described elsewhere [17, 19], autologous bone marrow (BM) tissue was aspirated using a commercial trochar and concentrated for MSCs under sterile conditions. Briefly, the posterior superior iliac spine was marked with ultrasound guidance for BM aspiration. Then 2% lidocaine was injected into the soft tissue and periosteum. An entry point was created using the introducer 14G trocar needle with which the bone was drilled through the periosteum and cortex and into the spongy bone. Using heparinized syringes, 1–6 cc were subsequently aspirated per level while slowly withdrawing until approximately 60 cc of aspirate was collected. BMAC was obtained and processed as described before for the mononuclear fraction containing MSCs (CD45^−^CD44^+^CD90^+^CD105^+^) among others [19].

### PRP preparation

PRP tissue was processed using the Harvest Technologies SmartPrep Multicellular Processing System (Terumo BCT, Inc., Lakewood, CO). First, 30 cc of blood was withdrawn from patients, anti-coagulated with acid citrate dextrose, and centrifuged for 14 min. Three blood layers were then obtained and the upper two, the plasma and the buffy coat, collected to obtain the final PRP product.

### Injection protocol

The injection site was prepared in sterile conditions using chlorhexidine swabs. Under ultrasound guidance, the area was visualized and marked, then PRP or BMAC injected into the appropriate knee intra-articularly. As seen in other studies (20,33), knees were injected unilaterally or bilaterally. In case of unilateral knee OA, the knee was injected 1–3 times within a one-month period at maximum depending on the grade of OA. For bilateral knee OA, both knees were injected simultaneously. The number and application of injections and the grade of OA are further detailed in the supplementary material per knee per patient per group (see Additional file 1: Tables 1–2).

### Baseline and follow-up measurements

In the baseline visit, all patients provided their date of birth and gender; were clinically assessed for range of motion (ROM), effusions, swelling, and tenderness to palpation at the joint line; and self-reported their knee-related pain and functionality using the three clinically validated questionnaires: the VAS, the KOOS, and the WOMAC. The first follow-up post-injection occurred after 2 weeks in the clinic. Subsequently, follow-up visits occurred at 1, 3, 6, 9, and 12 months and entailed clinical reassessments and remeasurements of knee-related pain and functionality scores as well as collection of treatment-emergent adverse events.

### Statistical methods

Baseline demographic and patient disease history information were summarized for the sample stratified by the numbers of knees injected and type of injection and presented as mean with standard deviation (SD) or 95% confidence interval (CI); median with interquartile range (IQR); or count with percent (%) according to the type and/or normality of variables. The Shapiro-Wilk test was used to verify the normality of data distribution. Knee groups were compared at baseline in all variables using Independent-Samples t-test, Mann-Whitney U test, Chi-Squared test, or Independent-Samples Median test based on the variable type and normality or non-normality of distribution.

The repeated-measures general linear model (GLM) with Sidak test for pairwise comparisons was used to investigate the influence of the treatment on the evolution of all knee-related clinical scores within a group. As such, time was considered a within-subject variable. The primary variable of interest within a group was the effect of time and the difference of estimated marginal means.

The repeated-measures GLM with Sidak test for pairwise comparisons was used to compare the change in VAS, KOOS, and WOMAC scores between knee groups throughout the duration of the study. As such, time was considered a within-subject variable and treatment a between-subject factor. The primary variable of interest between both groups was the effect of the treatment and the difference of estimated marginal means.

Pearson or Spearman correlation (2-tailed) was performed to assess correlation effects based on the types of variables.

All statistical analyses were performed using SPSS version 20.0 (IBM Corp). For all tests, *P* < 0.05 was considered significant.

## Results

### Baseline demographics and clinical characteristics

As seen in other studies [20, 33], data analyzed per patient were from either unilaterally or bilaterally injected knees. A total of 40 osteoarthritic knees (belonging to 30 patients) that have received either injection (BMAC, *n* = 27 vs PRP, *n* = 13) were assessed. One knee belonging to one patient treated with BMAC was lost to follow-up and thus could not be included in the analysis due to lack of complete data. No other serious complications or adverse events were recorded. Except for WOMAC scores, baseline parameters were homogeneously distributed between both groups (Table 2).

**Table 2:** Baseline demographics and clinical comparisons

Patient characteristics	BMAC (*n* = 26)^a^	PRP (*n* = 13)^a^	*P* value
Age, mean (SD)	58.46 (17.75)	53 (16.67)	.354^b^
Female, n (%)	12 (46.15)	6 (46.15)	1.00^c^
Left knee injections, n (%)	11 (42.3)	6 (46.2)	.82^c^
Degree of OA, n (%)	1, 8 (30.8%)	1, 2 (15.4%)	.35^c^
	2, 8 (30.8%)	2, 8 (61.5%)	
	3, 8 (30.8%)	3, 3 (23.1%)	
	4, 2 (4%)	4, 0 (0%)	
Number of treatments, median (IQR)	3 (1–3)	1 (1–3)	.424^d^
Total volume injected (ml), median (IQR)	24 (15–43.75)	8 (6–17.5)	.088^d^
Baseline knee-related clinical scores			
VAS, median (IQR) Baseline	6 (5–8)	4 (3–7)	.142^e^
KOOS, median (IQR)	44.5 (32.5–70)	67 (51.5–68.5)	.076^e^
WOMAC, median (IQR)	42.5 (37–74)	70 (50–73)	**.023**^e^

^a^Knees treated and analyzed

^b^Independent-samples t-test

^c^Chi-Square test (2-sided)

^d^Independent-samples median test

^e^Independent-samples Mann-Whitney U test

### BMAC provides more clinical benefits than PRP in patients with knee OA

**Fig. 1:**
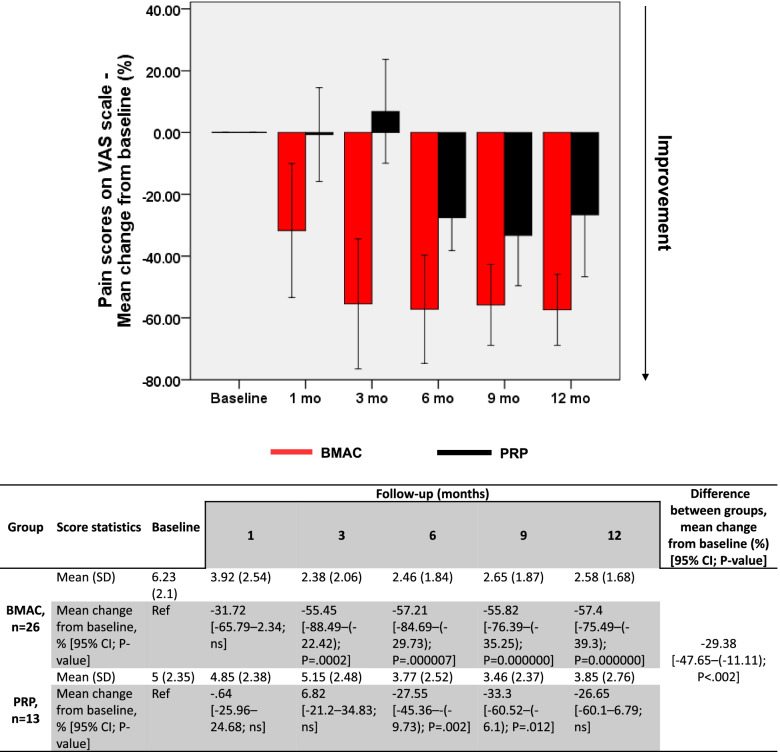
Evolution of VAS scores in BMAC and PRP groups. Changes (%) in VAS scores from baseline are represented as mean ± 95% CI. A general linear model for repeated measures was used to calculate *p*-values within each group compared to baseline and between treatment groups. BMAC, bone marrow aspirate concentrate; CI, confidence interval; ns, nonsignificant; PRP, platelet-rich plasma; Ref, reference

To compare the potency of both autologous treatments, BMAC- and PRP-treated patients were followed up on several parameters over 12 months following the procedure. A statistically significant improvement was observed in all clinical scores of BMAC-injected knees. More specifically, VAS scores improved by 57.4% on average, decreasing from 6.23 (±2.1) at baseline to 2.58 (±1.68) at 12 months (*P* = .000) (Fig. 1).

**Fig. 2:**
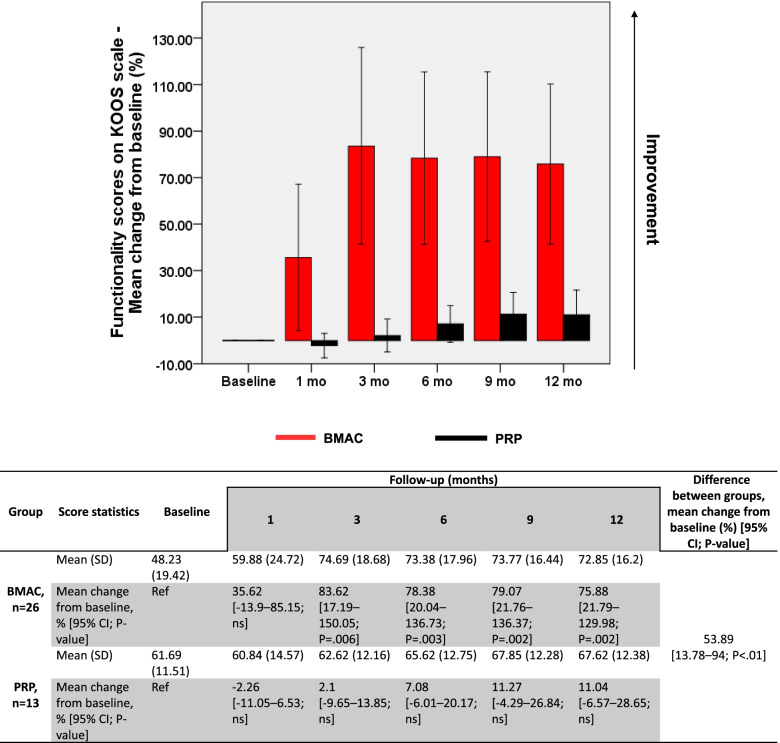
Evolution of KOOS scores in BMAC and PRP groups. Changes (%) in KOOS scores from baseline are represented as mean ± 95% CI. A general linear model for repeated measures was used to calculate p-values within each group compared to baseline and between treatment groups. BMAC, bone marrow aspirate concentrate; CI, confidence interval; ns, nonsignificant; PRP, platelet-rich plasma; Ref, reference

Similarly, KOOS scores improved by 75.88% on average, increasing from 48.23 (±19.42) at baseline to 72.85 (±16.2) at 12 months (*P* = .002) (Fig. 2).

Changes (%) in KOOS scores from baseline are represented as mean ± 95% CI. A general linear model for repeated measures was used to calculate *p*-values within each group compared to baseline and between treatment groups. BMAC, bone marrow aspirate concentrate; CI, confidence interval; ns, nonsignificant; PRP, platelet-rich plasma; Ref, reference.

**Fig. 3:**
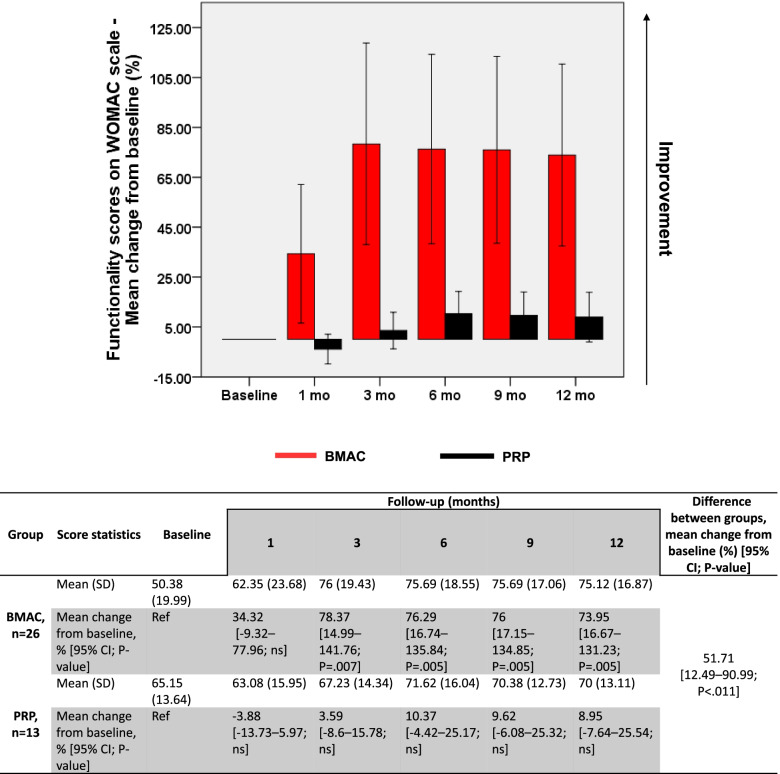
Evolution of WOMAC scores in BMAC and PRP groups. Changes (%) in WOMAC scores from baseline are represented as mean ± 95% CI. A general linear model for repeated measures was used to calculate p-values within each group compared to baseline and between treatment groups. BMAC, bone marrow aspirate concentrate; CI, confidence interval; ns, nonsignificant; PRP, platelet-rich plasma; Ref, reference

WOMAC scores also showed a significant 73.95% improvement on average, increasing from 50.38 (±19.99) at baseline to 75.12 (±16.87) at 12 months (*P* = .005) (Fig. 3).

On the other hand, no significant overall improvement was documented in any of the clinical scores assessed for PRP-injected knees. VAS scores fluctuated between baseline and 3 months around 5 (±2.35), then decreased significantly at 6 months by 27.55% (*P* = .002) and at 9 months by 33.3% (*P* = .012). At 12 months, however, VAS scores decreased to 3.85 (±2.76) albeit without attaining a significant difference compared to baseline (Fig. 1). KOOS scores also recorded a nonsignificant improvement between baseline (61.69 ± 11.51) and 12 months (67.62 ± 12.38) (Fig. 2). Similarly, a nonsignificant improvement in WOMAC scores was reported between baseline (65.15 ± 13.64) and 12 months (70 ± 13.11) (Fig. 3). Intergroup analysis showed significant differences between BMAC and PRP treatments in all adopted clinical scores, with mean improvements across all follow-up measurements being significantly higher in the BMAC group (Figs 1, 2, 3). Noteworthy, baseline WOMAC scores between both groups were significantly different, which could have biased the time-dependent intergroup statistical differences in this scale. Overall, these data show that BMAC treatment provides more clinical benefits to knee OA patients compared to PRP therapy.

### BMAC-induced improvements rely on distinct factors

**Fig. 4:**
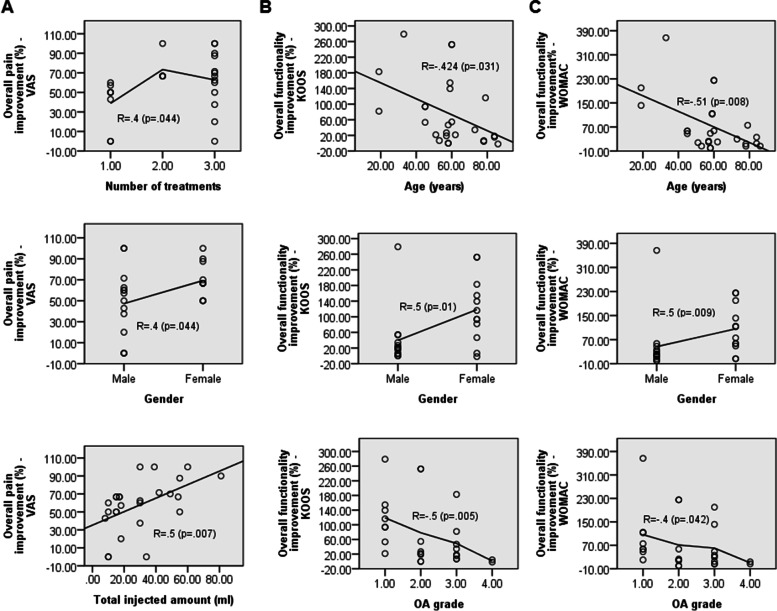
Factors correlated with score improvements following BMAC injection. Scatter plots with fit or interpolation lines displaying overall improvement (%) of BMAC-injected knees (*n* = 26) at 12 months under (**A**) VAS scores as a function of number of treatments received, gender, and total injected amount (ml) and (**B**) KOOS and (**C**) WOMAC scores as a function of age (years), gender, and baseline OA grade. R, Pearson’s or Spearman’s correlation coefficient

In the BMAC group, improvement in all scores correlated with patients’ gender, with female patients exhibiting better responses than their male counterparts. Furthermore, improvement in VAS scores was dose-dependent, meaning that the greater the number of treatments and BMAC volume, the better the pain score. Both KOOS and WOMAC scores were further age- and OA degree-dependent. This implies that improved functionality correlates positively with younger patients exhibiting milder OA symptoms (Fig. 4 and Table 3). In PRP-treated patients, improvements in all scores were highly dependent on the volume of injected PRP. Furthermore, improvement in VAS scores was positively correlated with male patients and was dose-dependent (Table 3).

**Table 3:** Factors correlated with the improvement in clinical scores in BMAC and PRP groups

Group	Improved outcomes	Correlated factors				
		Gender	Age	OA grade	Ascending number of treatments	Ascending injected amounts
BMAC, n = 26	**VAS**	Males<Females; R = .4 (*p* = .044)	ns	ns	R = .4 (*p* = .044)	R = .5 (*p* = .007)
	**KOOS**	Males<Females; R = .5 (p = .01)	Younger>Older; R = −.42 (*p* = .031)	Better grade > Worse grade; R = −.5 (p = .005)	ns	ns
	**WOMAC**	Males<Females; R = .5 (*p* = .009)	Younger>Older; R = −.51 (*p* = .008)	Better grade > Worse grade; R = −.4 (*p* = .042)	ns	ns
PRP, n = 13	**VAS**	Males>Females; R = .61 (*p* = .027)	ns	ns	R = .62 (*p* = .025)	R = .67 (p = .012)
	**KOOS**	ns	ns	ns	ns	R = .77 (p = .002)
	**WOMAC**	ns	ns	ns	ns	R = .73 (p = .005)

*R* Pearson’s or Spearman’s correlation coefficient, *ns* Nonsignificant correlation

### BMAC is more beneficial than PRP especially in milder OA grades

**Fig. 5:**
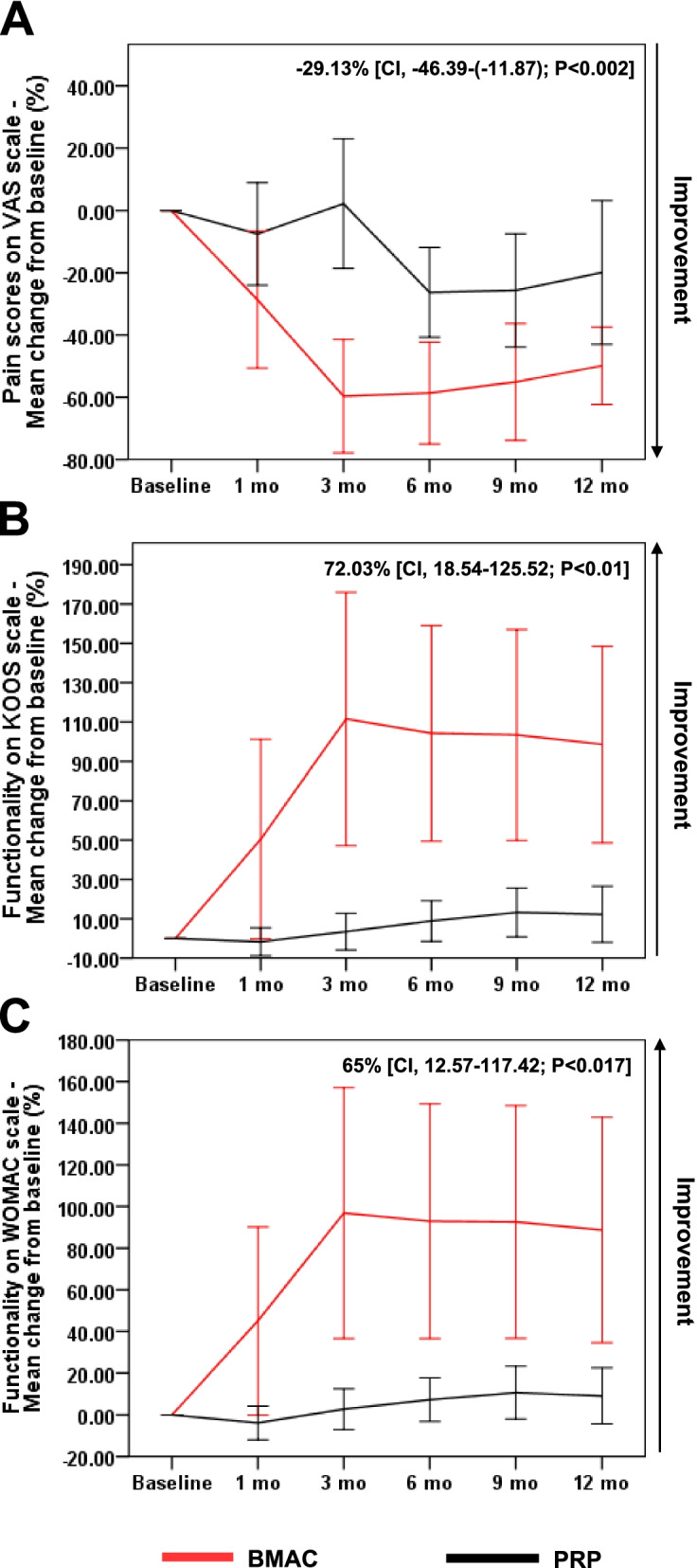
Evolution of clinical scores in patients with KL grades 1–2 between BMAC (*n* = 16) vs PRP groups (*n* = 10). Changes (%) in (**A**) VAS scores, (**B**) KOOS scores, and (**C**) WOMAC scores from baseline are represented as mean ± 95% CI. Mean % change differences between treatment groups across 1 year are provided as mean change from baseline (%) [95% CI; *P*-value]. A significant difference was recorded in the mean change from baseline (%) in all scores. A general linear model for repeated measures was used to calculate p-values between treatment groups. BMAC, bone marrow aspirate concentrate; CI, confidence interval; PRP, platelet-rich plasma

In subgroup analysis, we categorized patients into milder (grades 1-2) and more severe (grades 3-4) OA groups to further evaluate the effect of OA grades on clinical outcomes. The distribution of OA grades 1-2 vs grades 3-4 was homogeneous within treatment groups and between treatment groups (see Additional file 1: Table 3). Within treatment groups, no significant differences were recorded between OA grades 1-2 vs grades 3-4 in the improvement at 12 months in any of the 3 clinical scores (see Additional file 1: Fig. 1). Between treatment groups, however, significant differences were observed over 12 months in all adopted clinical scores only in patients with KL grades 1-2 (Fig. 5). Overall, our data show that BMAC treatment could be more beneficial than PRP in patients with knee OA, especially with milder disease grades.

## Discussion

Current knee OA treatment options, such as analgesics, corticosteroid injections, HA injections, and surgery, focus on managing joint pain and inflammation, yet their safety and long-term efficacy are still questioned [6–9, 34]. To bypass these issues, recent investigational studies have adopted regenerative medicine products with the rationale of treating OA pathology by impacting the knee joint homeostasis [10, 11, 23, 35]. PRP, a platelet- and growth factor-studded therapy, has gained considerable attention in clinical settings, demonstrating 1-year long benefits, especially in younger patients with mild knee OA [36, 37]. The therapeutic outcomes of PRP could be linked to platelet- or non–platelet-derived growth factors, such as insulin-like growth factor 1 (IGF-1) and platelet-derived growth factor (PDGF), which induce anabolic and structural changes at the level of the articular extracellular matrix and stem cell niche, and may attenuate degenerative pro-inflammatory processes [38]. Recently, a meta-analysis of 18 randomized controlled trials (RCTs) of knee OA has shown that PRP elicits better improvement in pain and functionality scores compared to HA [39]. Contrarily, another meta-analysis of 43 RCTs revealed no differences between PRP and HA in terms of ameliorating pain and functionality [40]. Considering these contradictory reports, evidence for PRP’s therapeutic superiority remains inconclusive. BMAC, on the other hand, harbors potent regenerative potential owing to its richness in platelets, white blood cells, and MSCs [12] that are characterized by their multi-lineage differentiation potential, anti-inflammatory profile, and pro-angiogenic auxiliary effects [16]. However, it remains unclear whether PRP and BMAC/MSCs lead to comparable outcomes in knee OA [23]. This knowledge gap formed therefore the basis of the herein investigation.

Although head-to-head comparisons with the literature are difficult to perform due to the relatively small number of studies investigating BMAC as a monotherapy in knee OA and the vast differences in study designs and measured outcomes [41], our results are generally in line with previous findings showing that BMAC significantly improves VAS [20, 33, 42], KOOS [20, 33], and WOMAC scores [43]. Notable, the statistical difference in WOMAC scores between both groups could have been biased due to the imbalance reported at baseline. The observed improvements correlated with several factors. More specifically, female patients receiving 2–3 treatments with higher volumes were more likely to improve on the pain scale compared to male patients receiving single treatments. Correlation data are also in line with previous studies demonstrating that the female gender, younger age groups, higher dose cellularity, and milder OA all have better prognosis [20, 33, 44–46]. In explanation of these correlated factors, increasing age was shown to be associated with reduced MSC numbers, lifespan, and proliferation/differentiation capacity [47–49]. In addition, human female MSCs were observed to divide more rapidly, exist in higher amounts in cell preparations, and promote a stronger anti-inflammatory environment by suppressing T-cell proliferation [50]. In terms of safety, although no BMAC-emergent adverse events were recorded, it is important to note the potential risks associated with bone marrow aspiration, including anemia, postoperative pain, neuralgia, and minor complications [51].

Although PRP-treated knees (*n* = 13) showed no statistically significant improvement on the scales of pain and functionality, the discrepancy between our results and previous data demonstrating remarkable clinical benefits with PRP [52–55] could be linked to our relatively small sample size preventing the detection of statistically significant improvement compared to baseline.

A recent study has examined the biologic differences between BMAC and PRP, showing that both differ in the concentration of leukocytes, cytokines, and growth factors, but not platelets, which might indicate potential therapeutic differences in orthopedic conditions [56]. Still and all, the two comparative analyses between BMAC and PRP in the literature have shown equivalent efficacy between both therapies with up to 12-month follow-up [23, 43]. A RCT was also conducted to determine which treatment is more effective, without yet disclosing any results (NCT03825133) [57]. Another trial has compared PRP-enhanced MSCs vs PRP, without disseminating further data (NCT01985633) [58]. The combined use of BMAC and PRP injected 1-to-2-month apart has shown to provide benefits in retrospective case series; however, the proof of synergism is unclear [59, 60]. In our study, we show that BMAC therapy results in more significant improvements in knee pain and functionality than PRP throughout 12 months of follow-up (Graphical abstract). The weight of these improvements is concentrated in milder knee OA subgroups (knee OA grades 1-2) (Fig. 5). Further follow-up is also expected to support these outcomes. Noteworthy, it is pivotal to highlight the small sample size of the PRP group as well as the fact that current protocols have no consensus in terms of the used methodology, including the preparation, dosage, and administration of PRP and BMAC, which could weaken the quality of data and pose a challenge for comparative analyses [18, 41]. Indeed, the differences in treatment protocols (ie, number of injections and volume injected) for each group, albeit balanced at baseline, could have impacted the quality of our intergroup outcomes. More homogenized treatment protocols are thus necessary to strengthen the evidence of intergroup differences. Additionally, another confounding factor potentially affecting intergroup differences is the effect of heparin used during BM aspiration. Recently, it has been shown that heparin impacts gene expression in BM-derived stromal cells without affecting their multilineage differentiation potential [61].

Overall, our results corroborate the therapeutic benefits of BMAC in patients with symptomatic knee OA (Graphical abstract). Notable, our study limitations remain its: i) small sample size, ii) differences in group sizes, iii) uncontrolled nature, and iv) lack of morphological evaluation of knee cartilages pre- and postoperatively. A larger RCT with more standardized operating procedures will thus be necessary to validate the efficacy of both investigated therapies and their equivalence or lack thereof. Whether higher numbers of treatments and treatment volumes or combined therapy could further improve clinical outcomes is also a potential future directive.

## Conclusion

Intra-articular autologous BMAC therapy safely and effectively reduced pain and improved functionality in patients with symptomatic knee OA to a greater extent than PRP therapy.

## Supplementary Information

**Additional file 1 **BMAC and PRP treatment modalities and OA grade-based distribution and sub-analyses. **Table 1** and **Table 2** demonstrate the data of both treatment modalities, BMAC and PRP, including patient ID, knees injected, knee OA grade, number of treatments received, and the volume injected per treatment. **Table 3** demonstrates the distribution analyses of OA grades 1-2 vs OA grades 3-4 within treatment groups and between treatment groups. **Figure 1** compares the improvement in clinical scores at 12 months between patients with OA grades 1-2 vs OA grades 3-4 within each treatment group.

## Data Availability

The datasets supporting the conclusions of this article are included within the article and its additional file.

## Abbreviations

BM: Bone marrow
BMAC: Bone marrow aspirate concentrate
CI: Confidence interval
GLM: General linear model
HA: Hyaluronic acid
IQR: Interquartile range
MSC(s): Mesenchymal stem cell(s)
OA: Osteoarthritis
PRP: Platelet-rich plasma
RCTs: Randomized controlled trials
ROM: Range of motion
SD: Standard deviation
