# Supplementary material for: Bone marrow aspirate concentrate versus platelet-rich plasma for treating knee osteoarthritis: a one-year non-randomized retrospective comparative study
Source: BMC Musculoskelet Disord. 2022 Jan 3;23:23. doi: 10.1186/s12891-021-04910-5 (PMC8725314; doi:10.1186/s12891-021-04910-5)
Supplement: Supplementary file 1 — Additional file 1 BMAC and PRP treatment modalities and OA grade-based distribution and sub-analyses. Table 1 and Table 2 demonstrate the data of both treatment modalities, BMAC and PRP, including patient ID, knees injected, knee OA grade, number of treatments received, and the volume injected per treatment. Table 3 demonstrates the distribution analyses of OA grades 1-2 vs OA grades 3-4 within treatment groups and between treatment groups. Figure 1 compares the improvement in clinical scores at 12 months between patients with OA grades 1-2 vs OA grades 3-4 within each treatment group. [file 12891_2021_4910_MOESM1_ESM.docx]

Additional File 1

**Table 1. BMAC treatment modality.**

| Patient ID | Knees injected (n=26) | OA grade | Number of treatments | Consecutive amount injected per treatment (ml) |
| --- | --- | --- | --- | --- |
| 1 | Knee R | 4 | 3 | 20, 15, 20 |
| 2 | Knee L | 3 | 3 | 10, 10, 10 |
|  | Knee R | 3 | 3 | 10, 10, 10 |
| 3 | Knee R | 1 | 3 | 9, 30, 10 |
| 4 | Knee L | 3 | 3 | 10, 9, 15 |
|  | Knee R | 4 | 3 | 10, 10, 10 |
| 5 | Knee R | 3 | 3 | 20, 20, 20 |
| 6 | Knee L | 2 | 1 | 15 |
|  | Knee R | 2 | 1 | 15 |
| 7 | Knee R | 1 | 3 | 16, 10, 16 |
| 8 | Knee L | 1 | 2 | 5, 10 |
|  | Knee R | 1 | 2 | 5, 10 |
| 9 | Knee L | 2 | 3 | 6, 6, 6 |
| 10 | Knee R | 2 | 1 | 18 |
| 11 | Knee R | 2 | 1 | 10 |
| 12 | Knee L | 2 | 1 | 10 |
|  | Knee R | 2 | 1 | 10 |
| 13 | Knee L | 3 | 3 | 20, 20, 15 |
| 14 | Knee L | 3 | 3 | 18, 18, 18 |
| 15 | Knee R | 2 | 3 | 10, 10, 10 |
| 16 | Knee R | 1 | 1 | 8 |
| 17 | Knee L | 1 | 2 | 7, 10 |
|  | Knee R | 1 | 2 | 7, 10 |
| 18 | Knee L | 1 | 1 | 10 |
| 19 | Knee L | 3 | 2 | 17, 22 |
|  | Knee R | 3 | 3 | 26, 35, 20 |

*Knee R, right knee; Knee L, left knee.*

**Table 2. PRP treatment modality.**

| Patient ID | Knees injected (n=13) | OA grade | Number of treatments | Consecutive amount injected per treatment (ml) |
| --- | --- | --- | --- | --- |
| 20 | Knee L | 3 | 5 | 4, 4, 4, 4, 4 |
|  | Knee R | 3 | 5 | 4, 4, 4, 4, 4 |
| 21 | Knee L | 3 | 1 | 4 |
| 22 | Knee L | 2 | 1 | 8 |
|  | Knee R | 2 | 1 | 8 |
| 23 | Knee R | 2 | 3 | 12, 12, 12 |
| 24 | Knee L | 2 | 1 | 15 |
| 25 | Knee R | 1 | 1 | 13 |
| 26 | Knee R | 2 | 1 | 4 |
| 27 | Knee R | 1 | 1 | 5 |
| 28 | Knee L | 2 | 3 | 4, 4, 4 |
| 29 | Knee L | 2 | 1 | 7 |
|  | Knee R | 2 | 1 | 7 |

*Knee R, right knee; Knee L, left knee.*

**Table 3. Distribution of OA grades within and between treatment groups.**

| Treatment group | OA grade, n (%) | Within-group *P* value* | Between-group *P* value** |
| --- | --- | --- | --- |
| BMAC (N=26) | OA grade 1–2, 16 (61.54%) | .239 | .278 |
|  | OA grade 3–4, 10 (38.46%) |  |  |
| PRP (N=13) | OA grade 1–2, 10 (76.92%) | .052 |  |
|  | OA grade 3–4, 3 (23.08%) |  |  |

*Chi-Square test (Fisher’s exact test).

**Chi-Square test.


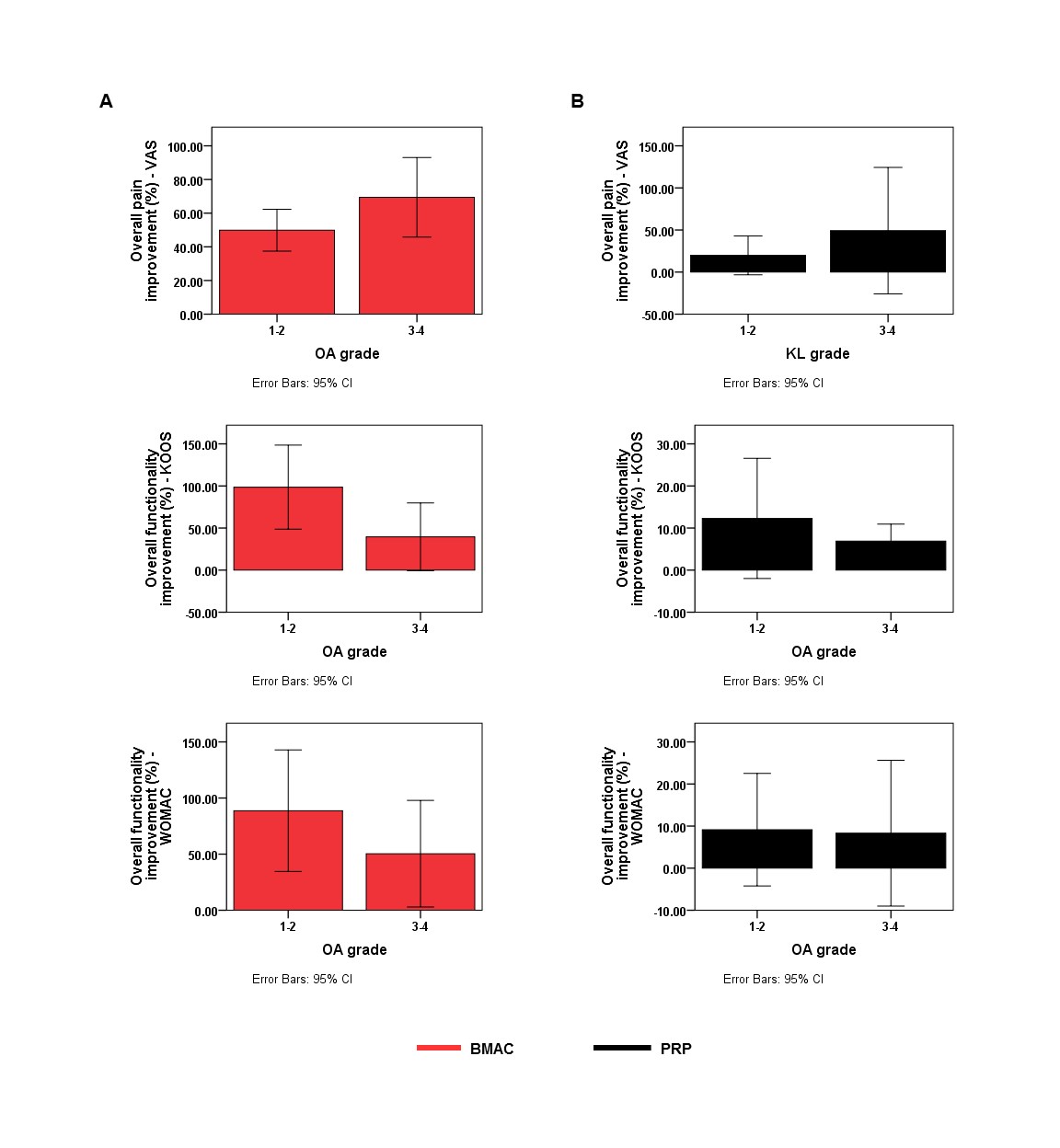


**Figure 1.** Improvement (%) in VAS, KOOS, and WOMAC scores at 12 months compared to baseline between patients with OA grades 1-2 and patients with OA grades 3-4 in (**A**) BMAC treatment group (n=26) and (**B**) PRP treatment group (n=13). No significant differences were recorded within treatment groups between both OA grade categories.
